# Family connections in the treatment of relatives of people with eating disorders and personality disorders: study protocol of a randomized control trial

**DOI:** 10.1186/s40359-023-01138-x

**Published:** 2023-03-30

**Authors:** Verónica Guillén, Antonio Arnal, Sandra Pérez, Joaquín Garcia-Alandete, Isabel Fernandez-Felipe, Antoni Grau, Cristina Botella, José Heliodoro Marco

**Affiliations:** 1 Universidad de Valencia, Facultad de Psicología, Departamento de Personalidad, Evaluación y Tratamiento Psicológico, Avda. Blasco Ibañez, 21, CP 46010 Valencia, Spain; 2 Ciber Fisiopatologia Obesidad y Nutrición (CB06/03), Instituto Salud Carlos III, Madrid, Spain; 3 Universitat Jaume I de Castelló, Facultad de Ciencias de la Salud, Avda Sos Baynat, S/N, Castellón de la Plana, Castellón Spain; 4 Ita-Salud Mental, Especialistas en Salud Mental, C/Tavern, 61, 08006 Barcelona, Spain

**Keywords:** Eating disorders, Personality disorders, Family connections, Caregivers, Dialectical behavior therapy

## Abstract

**Background:**

Eating disorders (EDs) are serious disorders that significantly affect not only the lives of patients, but also those of their family members who often experience high levels of burden, suffering and helplessness. If, in addition to ED, the patient has a personality disorder (PD), the psychological distress experienced by family members can be devastating. However, few treatments have been developed for family members of people with ED and PD. Family Connections (FC) is a programme that has been shown to be effective for family members of people with borderline personality disorder. The overall aims of this work are: (a) to adapt FC for application to family members of patients with BPD-PD (FC: ED-PD); (b) to analyse, in a randomised controlled clinical trial, the efficacy of this programme in a Spanish population, compared to a control condition consisting of treatment as usual optimised treatment (TAU-O); (c) to analyse the feasibility of the intervention protocol; (d) to analyse whether the changes that may occur in relatives are related to improvements in the family climate and/or improvements observed in patients; and (e) to analyse the perceptions and opinions of relatives and patients about the two intervention protocols.

**Methods:**

The study uses a two-arm randomised controlled clinical trial with two experimental conditions: adaptation of FC programme (FC: ED-PD) or Treatment as usual optimised (TAU-O). Participants will be family members of patients who meet DSM-5 criteria for ED and PD or dysfunctional personality traits. Participants will be assessed before and after treatment and at one-year follow-up. The intention-to-treat principle will be used when analysing the data.

**Discussion:**

The results obtained are expected to confirm the effectiveness of the programme and its good acceptance by family members.

*Trial registration* ClinicalTrials.gov Identifier: NCT05404035. Accepted: May 2022.

## Background

Eating disorders (ED) produce significant disturbances related to food intake, and they often result in intense preoccupation with weight and the figure, as well as the use of control strategies such as dieting and purging [1]. In severe cases or without adequate treatment, ED can affect the body’s organs [2] and the individual’s social functioning, and they are associated with psychological problems such as depression, anxiety, social isolation, family conflicts, low self-esteem, a negative self-concept, and reduced autonomy [3].

A diagnosis of an ED is a burden for families or primary caregivers (usually mothers) that impacts their daily lives. Caregivers of patients with ED commonly experience mental health problems, psychological distress, and burden due to their caregiving experiences, which affects their mental health, quality of life, and well-being [4–6]. Caregivers frequently express the need for information about how to help their loved one with ED recover from the disorder [7]. Thus, it is necessary to include family members in treatment programs that offer support and psychoeducation [8].

So far, there are three types of empirically supported interventions for family members of people with eating disorders: (a) psychoeducational interventions [8–11], (b) interventions based on systemic cognitive behavioural therapy [12–14], and (c) interventions based on the New Maudsley Model [15–22].

Sepúlveda et al. [23] conducted the first RCT with Spanish ED caregivers to study the effectiveness of The Collaborative Care Skills Workshop program by comparing it with a psychoeducational group program containing six two-hour sessions based on Fairburn’s [24] program. Patients were receiving parallel CBT for ED. The results indicated that, although there were no statistically significant differences between the two experimental conditions, after treatment there was an improvement in the relatives’ perception of illness, adaptation to ED symptoms, perceptions of themselves as caregivers, and level of distress. However, no statistically significant differences were found between before and after treatment in the level of expressed emotion, emotional over-involvement, critical comments, family adaptation to the disorder, anxiety, depression, self-efficacy, experience of caring, or impact of ED symptoms on family members. Regarding the patients, there was a reduction in their symptoms of ED and psychological distress after the intervention, but it was not maintained at follow-up. Sepúlveda et al. [23] suggested that six sessions could be insufficient to find changes after treatment. In addition, it should be noted that the patients who participated had a long duration of the problem and were resistant to treatment, and although it was not evaluated, they might have had a comorbid personality disorder.

In summary, there is scientific evidence supporting the efficacy of interventions specifically designed to help caregivers of people with ED. All these programs focus on providing caregivers with knowledge about the functioning of the disease and strategies to provide adequate support for their loved ones while attending to their own needs. Overall, the results showed significant decreases in caregivers’ experienced burden, distress, anxious and depressive symptomatology, expressed emotion, and symptom accommodation behaviors. Likewise, there was a significant increase in self-efficacy, general well-being, and knowledge and skills in relating with their loved ones. In addition, some of the studies [10, 14, 16, 21, 23] also evaluated the effects of the program on the patients, generally reporting improvements in the progression of the disorder and a reduction in symptomatology. However, these results were not maintained at the follow-ups.

Some of the most important transdiagnostic symptoms of ED (impulsivity, emotional dysregulation, intolerance to emotions, and interpersonal problems) [25] are characteristic of Borderline Personality Disorder (BPD). This comorbidity involves a high frequency of binge eating, purging, difficulties in interpersonal relationships, risk of self-harm and suicide, non-adherence to treatment, and non-compliance with therapeutic tasks, all of which increases the risk of chronicity [26, 27]

Guillen et al. [28] conducted an intervention based on the principles of Dialectical Behavior Therapy (DBT) [29] with 115 family members of patients with a diagnosis of ED and PD who were undergoing treatment in a specialized unit. The intervention took place face-to-face and in groups, and it consisted of eight weekly sessions. The intervention modules were: psychoeducation about ED and PD, description of the emotional dysregulation model of PD, validation techniques, radical acceptance techniques, knowing the limits in the family environment, and learning to manage the characteristic problems of ED and PD (binge eating, vomiting, self-harm, suicidal behaviors, emotional outbursts, substance abuse, and relapses). The intervention was shown to be effective in reducing burden and increasing caregiver self-efficacy. Although results from this study can be considered preliminary because there was no control group, the results indicate that it is possible to help caregivers of people with ED and PD.

Psychoeducation groups for relatives of people diagnosed with BPD provide information about the illness and, thus, help them to understand some of the behaviors of their family members, in order to improve the relationship and family coexistence [30–33]. Fruzzetti’s group developed and tested the program *Family connections: A program for relatives of persons with Borderline Personality Disorder* (FC) [34], which is designed to be delivered either by professionals or by relatives who have previously completed a training course. The FC program contains 12 two-hour weekly sessions. The content of the intervention program is divided into six modules and includes: psychoeducation about BPD, how it affects family functioning, skills adapted from DBT [29] (individual, family, and relational skills, validation exercises, and problem-solving skills), and peer support. All the modules include specific practical exercises and homework assignments. In addition, throughout the FC program, there is a forum where participants can build a support network. To test the efficacy of FC, five uncontrolled clinical trials have been conducted so far, with pre-post evaluation moments and follow-ups [34–38]. The results of these five studies show significant decreases in family members’ subjective experience of illness burden, perceived distress, depression, and grief, as well as improved coping strategies. These changes were maintained at the three-month follow-up.

Despite the results from programs focused on the specific symptoms of ED, mentioned above, the studies did not report information about comorbid diagnoses, presence of self-harm, suicidal behaviors, or participants’ diagnosis of PD. Therefore, interventions with caregivers on these common symptoms, which are frequently transdiagnostic, have not been contemplated. Taking into account the high comorbidity observed in ED patients, it would be necessary to evaluate, diagnose, and adapt family support programs to include specific modules that help relatives to cope with the relationship with the patient, in addition to the specific symptoms of ED. Although one of the main objectives of the programs is to improve expressed emotion, none of these studies evaluated or focused on caregivers’ emotional regulation skills. Finally, these programs do not address the management of negative emotions stemming from grief about caring for a person with ED, and they do not usually assess caregivers’ quality of life. Thus, an adaptation of the Family Connections program for relatives of patients with an ED diagnosis would be highly beneficial.

### Objectives and hypotheses

The general objectives of this research project are: (1) to adapt and test the modules of the FC intervention protocol designed specifically for family members of patients with ED-PD in the Spanish population (FC: ED-PD); (2) to analyze, in a randomized controlled trial, the efficacy of the FC: ED-PD program, versus a control condition consisting of optimized treatment as usual (TAU-O), in reducing objective and subjective illness burden, possible clinical symptomatology, and hostility or emotional discomfort, as well as improving family climate and quality of life. The TAU-O control condition will consist of the usual treatment that each family member receives/chooses, depending on their situation, plus a psychoeducation component about ED; (3) to analyze the feasibility and acceptability of this intervention protocol in relatives of patients with ED-PD in a pilot feasibility study that takes different aspects into account: from acceptance by the participants through the consideration of legal and technical aspects, as well as the preparation and implementation of the intervention; (4) to analyze whether the changes that may occur in family members with respect to disease burden and clinical symptomatology are related to improvements in the family climate and/or improvements observed in the patients with ED-PD; and (5) to analyze the perceptions and opinions of families and patients about the two intervention protocols.

The general initial hypothesis, based on the results obtained by Guillén et al. [28] with relatives of patients with ED-PD and those obtained by Fruzzetti’s group in the population with PD [34, 36, 37, 39], is that the FC program designed and developed by Fruzzetti’s group will be effective (both statistically and clinically), compared to optimized treatment as usual (TAU-O), and efficient. The following specific hypotheses are proposed: (1) The FC: ED-PD protocol will be viable, i.e., it can be administered to relatives of patients with ED-PD in clinical centers, both in the National Health System and in private centers, and it will be well accepted by relatives, patients, and clinicians. (2) The FC: ED-PD protocol condition will be more effective than the control condition, which uses the optimized treatment as usual (TAU-O) participants may receive depending on their situation, but optimized with a specific psychoeducation component on ED-PD and its repercussions. (3) Family members, patients, and clinicians will rate the FC: ED-PD protocol significantly higher than the TAU-O control protocol. (4) The improvements that may occur in family members with respect to disease burden and clinical symptomatology will be related to improvements observed in patients with ED-PD and in the perceived family climate.

Attending to and training family members of patients with ED-PD in the different skills required to effectively manage these patients, both in daily life and in times of crisis, will help to alleviate the stress and burden that family members experience on a daily basis. This, in turn, will help to improve family relationships and patient outcomes.

The assessment of the program’s effectiveness for participants will be determined by their differential scores before versus after the application of the interventions and at the 12-month follow-up on the evaluation instruments used. Specifically, we expect: (a) a decrease in scores on variables measuring clinical symptomatology and disease burden (objective and perceived); (b) less interference of the problem in the lives of family members; (c) an improvement in perceived family climate; (d) decreased hopelessness; (e) improved coping strategies; (f) increased quality of life scores; (g) reduced intrafamily conflict; and (h) fewer visits to hospital emergency rooms and fewer emotional and behavioral outbursts by the patient.

The evaluation of the efficiency of the program will be determined by: (a) the ratio of family members who are offered the chance to participate in the program, compared to those who agree to participate; (b) the dropout rate of family members; (c) the evaluation of the program by family members who participate; (d) the evaluation of the program by clinicians who are introduced to the program and the demonstration of its feasibility; and (e) the evaluation of the program by clinicians who participate in the program and the demonstration of its feasibility. In this article, we present the study protocol.

## Methods

### Participants

The sample will be composed of relatives of patients who meet DSM-V criteria (1) for Anorexia Nervosa (both restricting and purging subtypes), Bulimia Nervosa, Binge Eating Disorder, Eating or Food Intake Disorder Not Otherwise Specified, and any PD (or PD traits). The diagnoses will be carried out by a specialist in clinical psychology or psychiatry, and the sample will be recruited in different clinical services: the University Clinical Hospital of Valencia and its Mental Health Reference Units; and the Eating Disorders and Personality Disorders Unit PREVI-ITA and its three centers in Castellón, Valencia, and Alicante.

In the case of the patients, the following inclusion and exclusion criteria will be established: 1) meet the diagnostic criteria for ED and PD (or PD traits); 2) agree to participate in the study in writing by signing the informed consent form; in the case of minors, the consent must be signed by their parents; 3) The presence of another serious pathology such as psychosis, schizophrenia, intellectual disability, etc., will be an exclusion criterion.

In the case of family members, the following inclusion and exclusion criteria will be followed: (1) being a family member of one of the patients with a diagnosis of ED and PD (or PD traits); (2) signing the informed consent; (3) The presence of any pathology in the family member that keeps the intervention from being carried out (such as psychosis, schizophrenia, intellectual disability, substance dependence, etc.) will be an exclusion criterion.

### Study design

First, a pilot feasibility study will be carried out using focus groups to explore the opinions of relatives of ED-PD patients. Semi-structured interviews will be used. The research design will follow the criteria established by Cooke et al. [40], and for the development of the focus groups, the guide developed by Breen [41] for this type of methodology will be used. Second, a randomized controlled clinical trial will be conducted following the CONSORT (Consolidated Standards of Reporting Trials) guidelines http://www.consort-statement.org/ [42, 43]. This study is a superiority trial. The study design consists of a two-arm randomized controlled trial (RCT). On the one hand, there will be two conditions: Family Connections (FC-ED—PD) or Treatment as usual optimized (TAU-O), and family members will be randomized to one of the two groups. -A between-subjects design will be used with three assessment points: pre-treatment, post-treatment, and a follow-up 6 and 12 months after the end of treatment. Once the study has been explained and the informed consent has been signed, participants will be randomly assigned to one of two experimental conditions: 1) The adaptation of the FC protocol for relatives of ED-PD patients (FC: ED-PD) will be applied; 2) The Treatment as Usual Optimized Treatment (TAU-O) protocol will be applied. Randomization will be performed by an external researcher who will not participate in any of the phases of the project. The randomization sequence will be hidden from the evaluators participating in the study. The G*Power 3.1 software [44] will be used for this purpose. Assessment of participants will be carried out by a clinician other than the professional administering the treatments. Upon completion of the randomized controlled clinical trial, in-depth interviews will be conducted with family members who have successfully completed the intervention (e.g. clinically significant change) and with family members who drop out of the intervention. In all the potential groups, the aim will be to explore participants' experiences in order to learn about barriers and facilitators of the intervention.

### Sample size

**Fig. 1:**
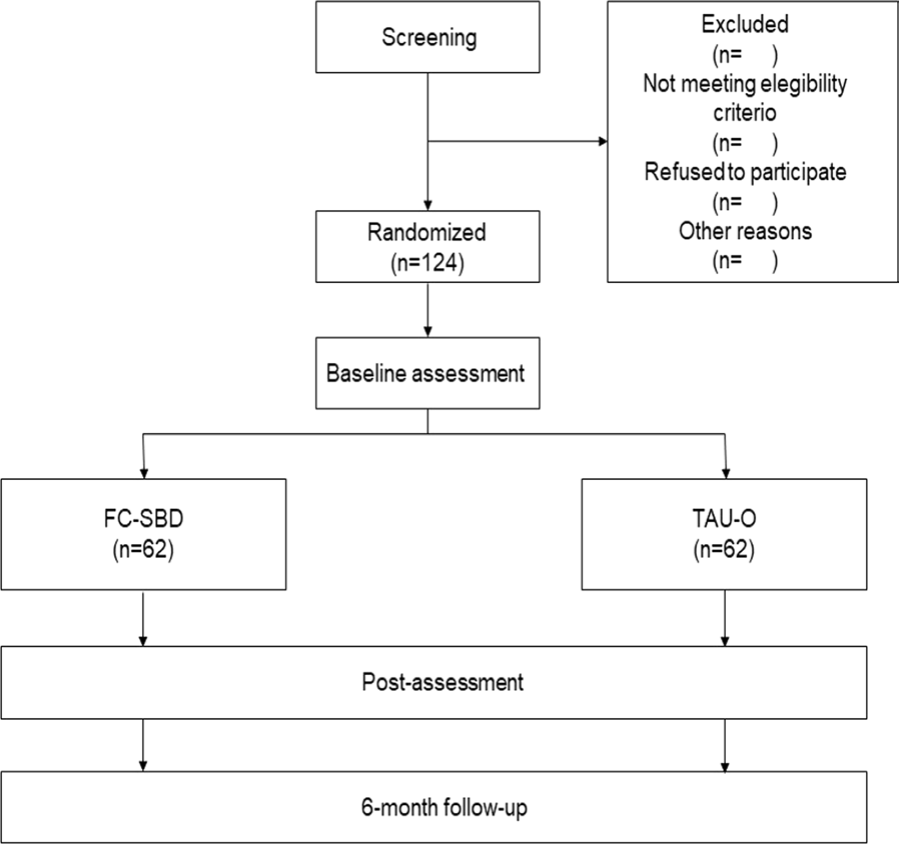
Flow Chart

To calculate the sample size, effect sizes found in previous studies on the topic have been considered. Grenyer et al. [30] tested a group psychoeducation protocol for caregivers of people with BPD in a controlled study that reports medium to large effect sizes (dyadic adjustment, *d* = 0.78; family empowerment, *d* = 1.4). Moreover, Grenyer obtained significant improvements on measures of illness burden between post-assessment and 12-month follow-up, with medium effects (*d* = 0.45). These effects are in line with the literature on psychological treatments for relatives of people with ED (Positive Caregiver Experience, *g* = -0.80) [14]. Given these data, an effect size of 0.50 is expected in the present study, adopting a conservative approach. Because our design includes two experimental conditions, t-tests are assumed for the statistical analyses. Considering an alpha of 0.05 and a statistical power of 0.80 in a two-tailed t-test, the total sample size needed to reach an effect size of 0.60 in loading is 90 participants (45 participants per experimental condition). To control for the maximum possible loss of subjects during treatment, based on the literature on previous programs for family members of BPD or ED patients, a dropout rate of 30% is expected [34–36]. Therefore, the required sample size should contain 124 participants in all (62 participants per group), as Fig. 1 shows. These calculations were carried out using the software program G*Power 3.1 [44].

### Procedure

Family members of patients with ED-PD will be given the opportunity to participate in the study. Once they have signed the informed consent, an expert clinician will perform the assessment of each participant to check that the inclusion and exclusion criteria are met, and an independent investigator, unaware of the characteristics of the study, will be contacted to perform the randomization. At all times, for randomization purposes, an experimenter from outside the research team will assign each family member to one of the two study conditions (FC: ED-PD or TAU-O) using a random number software program. The randomization sequence will be concealed from the clinicians participating in the study. Independent assessors will administer the assessment protocol to the participants without being aware of the experimental condition to which they belong (FC: ED-PD or TAU-O). Patients will also be assessed before starting the intervention programs for relatives. After the assessment has been completed, all the relatives will start to receive the intervention in the condition to which they have been assigned, and the patients will participate in their current treatment or care. At the end of the intervention, the assessment protocol will be re-administered to both relatives and patients, again by independent assessors, and the same thing will be done at the 12-month follow-up.

### Ethics

All methods will be conducted in accordance with the ethical standards of the declaration of Helsinki accordance with relevant guidelines and regulations. Researchers adhere to the Helsinki Convention and the World Psychiatric Association's Madrid Declaration on clinical research. All participants will be volunteers who have given their informed consent to participate in the study. All eligible participants will be given oral and written information about the study and the two intervention modalities. Specifically, they will be informed that they may leave the study at any time without giving an explanation, and that this decision will in no way affect their family member's regular treatment at the center. The selection and evaluation of the participants will be performed by qualified personnel who will not know to which condition a given participant has been assigned, and the treatments will be carried out by qualified and expert professionals. The Ethics Committee of the University of Valencia (Valencia, Spain) approved this study with number UV-INV_ETICA-1955599. The trial was registered at clinicalstrial.gov as NCT05404035, June 3rd 2022.

### Interventions

#### Family connections for relatives of people with eating disorders and personality disorders (FC: ED-PD)

The intervention lasts three months and includes 12 sessions with a weekly two-hour group format. The FC program [34]) is divided into six modules, but the research team developed the program adapted to ED and PD in seven modules, which are described below [34]:

Module 1: Presentation of the program, objectives, structure, tasks, etc. Up-to-date information and research on ED and PD (Epidemiology, frequency, Risk factors, and protective factors).
Module 2: Psychoeducation on the development of ED and PD, explanatory theories, available treatments, and comorbidity with PD.
Module 3: Emotional regulation skills, skills of acceptance, validation, approach, and awareness, and to decrease emotional reactivity.
Module 4: Skills to improve the quality of relationships in family interactions (letting go of guilt and anger, acceptance skills in relationships).
Module 5: Communication skills and effective self-expression.
Module 6: Problem management and making safe plans for crisis management.

All the modules include practice exercises, video viewing, and homework assignments. In addition, throughout the program, with the goal of increasing social support, the FC program provides a forum where participants can stay in touch and share common problems and solutions.

#### Optimized treatment as usual (TAU-O)

Family members in this condition will continue to receive the usual treatment provided by their referral care center. In addition, we will optimize the treatment they are currently following, based on the recommendations of the international guidelines for the treatment of ED [45]. There will be one three-hour session in group format, with the following components:

Module 1: Up-to-date information and research on ED and PD (epidemiology, frequency, risk factors, protective factors). Psychoeducation on the development of ED and PD. Explanatory theories. Available treatments and comorbidity.

In both conditions, after each face-to-face session, the participant will be asked to review the contents addressed during the session as homework (independently of the homework corresponding to the specific module being addressed). All the interventions with family members will be performed by clinical psychologists or general health psychologists with at least a master's degree or a doctoral degree, and with previous training in administering the programs.

#### Treatment of patients

With regard to the patients’ treatment, the routine treatment they receive in their centers of reference will be followed. All the interventions will be carried out by the clinical psychologists and psychiatrists working in these centers.

## Measures

### Family members’ measures (participants)

#### Primary outcomes

*Record of critical family-patient incidents* number of Binge eating and vomiting (purging) episodes in the past three months, number of serious arguments between patient and caregivers in the past three months, number of days of self-injury in the past three months, number of episodes of verbal/physical violence with caregivers in the past three months, number of visits to the psychiatric emergency department in the past three months, number of unscheduled therapy sessions in the past three months (face-to-face, phone calls, etc.).

*Burden assessment scale* [46]: It is a 19-item scale that assesses caregivers’ objective and subjective burden due to their loved one’s illness within the past six months. Items are rated on a 4-point Likert scale ranging from 1 (nothing) to 4 (a lot). Higher total scores indicate stronger burden. The scale shows adequate validity and reliability (Cronbach’ alpha ranges from 0.89 to 0.91) [47]

#### Secondary outcomes

*The eating disorder symptom impact scale* [48] () in its Spanish version (Carral‐Fernández et al., 2013). It is a 24-item scale that assesses eating disorder-specific caregiving experiences. Items are rated on a 5-point Likert-type scale ranging from 0 (never) to 4 (almost always). The internal consistency is good, with Cronbach’s alpha coefficients mostly superior to 0.70.

*Family assessment device—global functioning scale* [49]). It is a 60-item self-report about family functioning in terms of problem-solving, communication, roles, affective responsiveness, affective involvement, behavior control, and general functioning. Items are rated on a 4-point Likert scale ranging from 1 (completely agree) to 4 (strongly disagree). The internal consistency is good (Cronbach's alphas between 0.72 and 0.83) for the subscales, with a Cronbach's alpha of 0.92 for general functioning.

*Mastery and empowerment scale* [50]. It is a 34-item scale divided into three domains: family, service system, and involvement in the community. Items are rated on a 5-point Likert scale ranging from 1 (completely false) to 5 (completely true). Different studies have demonstrated that the psychometric properties of the FES are robust in both its original and translated versions [50–52].

*Multicultural quality of life index* [53]. The Multicultural Quality of Life Index is a culture-informed, self-rated instrument. Its 10 items cover key aspects of quality of life, from physical well-being to spiritual fulfilment. Regarding its applicability, the mean completion time was less than three minutes, and 96% of raters found it easy to use. Test–retest reliability was high (r = 0.87). A Cronbach’s alpha of 0.92 showed its internal consistency, and a factor analysis revealed a strong structure.

*The multidimensional existential meaning scale* [54] in its Spanish validation [55]. It is a 15-item scale that assesses existential meaning through three dimensions: comprehension, purpose, and mattering. Items are rated on a 7-point Likert scale ranging from 1 (very strongly disagree) to 7 (very strongly agree). The three MEMS subscales showed adequate internal consistency: Comprehension (ω¯ = 91), Purpose (ω¯ = 92), and Mattering (ω¯ = 86).

*Depression, anxiety and stress scale* [56]. It is a 21-item scale, in its short version, that measures clinical symptoms such as depression, anxiety, and stress. Items are rated on a 4-point Likert scale ranging from 0 (It did not happen to me) to 3 (It happened to me a lot, or most of the time). It shows excellent internal consistency: depression (α = 0.94), anxiety (α = 0.87), and stress (α = 0.91).

*Difficulties in emotion regulation scale* [57]. in its Spanish version [58]. In its adaptation, the authors reduced the items from 36 to 28, and they considered five scales instead six. The subscales are: lack of emotional control, vital interference, lack of emotional attention, emotional confusion, and emotional rejection. Items are rated on a Likert-type scale ranging from 1 to 5 (1 = "almost never" and 5 "almost always"). Higher scores indicate more difficulties in emotional regulation. Psychometric properties were adequate, with very good internal consistency (α = 0.93) and good test–retest reliability (pl = 0.74, p < 0.001).

#### Other pre-specified outcome measures

*Socio-demographic data* age, sex, educational level, income, marital status, number/age of children, and history of psychological treatment.

*Opinion of treatment scale,* adapted from Borkovec and Nau [59].

### Measures‑patients

#### Primary outcomes

*The eating attitudes test‐26* [60]. in its Spanish version [61]. *This scale is used to evaluate the patients*. It is a 26-item self-report that assesses attitudes toward eating. Items are rated on a 6-point Likert scale ranging from 1 (never) to 6 (always). The reliability analysis indicated good internal consistency [60, 62–65].

*Patient health questionnaire* [66]. in its Spanish version [67]. *This scale is used to evaluate the patients*. It assesses each of the nine DSM-IV criteria for depression through nine items. Items are rated on a 4-point Likert scale ranging from 0 (not at all) to 3 (nearly every day).

#### Secondary outcomes

*Overall anxiety severity and impairment scale* [68]. in its Spanish version [69]. *This scale is used to evaluate the patients.* It is a 5-item scale that assesses the severity and frequency of anxiety symptoms, behavioral avoidance, and the functional impairment related to anxiety. Items are rated on a 4-point Likert scale ranging from 0 to 4. It shows good psychometric properties in terms of test–retest reliability and internal consistency (α = 0.80).

*Validating and Invalidating responses scale* [70]. *This scale is used to evaluate the patients*. It is a 16-item scale that assesses the levels of perceived validation and invalidation of caregivers’ responses, divided into validating and invalidating responses. Items are rated on a 5-point Likert scale ranging from 0 (never) to 4 (almost always). Higher scores indicate greater perceived validation or invalidation of the responses of the caregiver being assessed.

*Lum emotional availability of parents* [71]. *This scale is used to evaluate the patients*. It is a 15-item questionnaire that assesses the emotional availability of mothers and fathers as perceived by the person assessing their relatives. Items are rated on a 6-point Likert scale ranging from 1 (never) to 6 (always). The psychometric properties are very good; for a clinical sample, excellent internal consistency is observed (for mother α = 0.92, and for father α = 0.93). In addition, it has adequate test–retest reliability for the mother's form (r = 0.92) and the father's form (r = 0.85).

### Data analyses

Regarding data analysis, the results of the pilot feasibility study using focus groups will be analyzed using thematic analysis in order to identify the topics that emerge from the focus groups [72]. Data reporting will be carried out following the COREQ guidelines [73]. In addition, qualitative research quality criteria recently developed by Levitt et al. [74] will be pursued in order to achieve what the authors refer to as methodological integrity in the qualitative field.

In the controlled clinical trial, the CONSORT guidelines [43] will be followed. First, participants' scores in the two conditions will be compared before the intervention to check that there are no significant differences between them on the outcome measures and that they are, therefore, comparable after randomization. ANOVAs will be conducted for continuous variables and Chi-square tests for categorical variables. For outcome measures at post-treatment, we will study whether the assumption of homoscedasticity is met with Levene's test. If this assumption is met, repeated-measures ANOVAs and F-tests will be used to compare the two experimental conditions. If the homoscedasticity assumption is not met, the Brown-Forsythe test will be applied. F-tests for statistical significance will be followed by post hoc comparisons. In particular, Tukey will be used when the homoscedasticity assumption is met, and Games-Howell if the homoscedasticity assumption is not met. Appropriate analyses will also be carried out to calculate intervention effect sizes and confidence intervals. The intention-to-treat principle will be used when analyzing pre- and post-treatment data and at 12-month follow-up, using mixed-effects models with full information and maximum likelihood estimation. To complement the results of ANOVAs and post hoc comparisons, effect sizes will be calculated using the standardized mean difference proposed by Cohen [75]. These effect sizes will be calculated to assess changes within and between groups, all based on a pooled standard deviation.

For the qualitative study, semi-structured in-depth interviews will be used. These interviews will follow the guidelines of Knox and Burkard [76]. The research design will be carried out following the criteria established by Cooke, Smith and Booth [40]. Data will be analyzed using the consensual qualitative research (CQR) method. Data reporting will be carried out following the COREQ guidelines [73]. Furthermore, we will attempt to meet the criteria for quality in qualitative research recently developed by Levitt et al. [74] in order to pursue what the authors refer to as methodological integrity in the qualitative field.

## Discussion

Family caregivers of patients with an ED diagnosis and a comorbid condition suffer from burden experiences, mental health problems, psychological distress, poor quality of life, and low wellbeing [4–6]. In addition, they express a need for information about the disorder and strategies to cope with it [7]. To date, programs for relatives of patients with a diagnosis of an ED have mainly focused on psychoeducation and support related to ED and cognitive-behavioral techniques [8, 9, 14]. However, they do not address ED comorbidities such as BPD symptoms, suicide and non-suicidal self-injuries, and impulsiveness, or focus on improving the relationship with the patient or on participants’ emotional regulation skills. The FC program has been found to be effective in decreasing relatives’ subjective experience of illness burden, perceived distress, depression, and grief, and improving their coping strategies. However, there are no RCTs that have demonstrated the efficacy of FC in relatives of patients with a diagnosis of an ED and PD or PD traits. For this reason, in this study, we have adapted some of the modules of the original FC program [34] to this population.

The first and second objectives of this work were to adapt and test the modules of the FC intervention protocol for family members of patients with ED-PD in the Spanish population and analyze, in a randomized controlled trial, the efficacy of FC ED-PD. For the last objective, we have designed an RCT that compares the FC for ED-PD program to a control condition consisting of treatment as usual (TAU-O), optimized with a component of psychoeducation about ED. The samples are recruited from different public and private centers specialized in the treatment of patients with an ED-PD diagnosis.

The third objective is to analyze the feasibility and acceptability of the FC intervention in participants, given that the participants’ acceptance of the program is as important as the reduction in symptomatology and the improvement in personal and social functioning. We consider aspects such as legal and technical issues, as well as the preparation and implementation of the intervention.

Our fourth objective is to analyze whether the changes related to perceived burden and clinical symptoms that may occur in relatives of patients with ED-PD are related to improvements in the family climate and/or improvements observed in patients with ED-PD. To our knowledge, previous studies on FC have not explored this relationship, except one previous proposal from our research team that consisted of an RCT that explored the benefits of FC in relatives of patients with suicide behaviors, although there are still no published results from this study [77]. Our last objective was to analyze the perceptions and opinions of families and patients about both intervention protocols.

This is the first RCT to test an adaptation of FC for relatives of patients with a diagnosis of ED-PD. Until now, previous studies have focused on the effectiveness of FC in relatives of patients with a BPD diagnosis [36, 39] and suicide behaviors [77, 78] (Marco et al., 2022; Rajalin et al., 2009), but no previous studies or RCTs have explored the effectiveness of FC in relatives of patients with an ED-BPD diagnosis. The present proposal emphasizes the transdiagnostic utility of the FC program and the important role of working on aspects such as reducing burden and learning emotion regulation, communication, and validation skills in improving the family climate, by adding the specific psychoeducational components related to ED-PD.

In addition, this study explores the efficacy of FC in Spanish participants. Previous studies have mainly explored the usefulness of FC in English-speaking relatives [34–38], and only some of them have focused on Spanish-speaking participants [28, 55, 77]. Thus, this work extends the knowledge about the usefulness, efficacy, and acceptability of the program in non-English speaking countries.

Finally, the present RCT compares FC to TAU-O in relatives of ED patients. The majority of studies with relatives of ED patients have proposed family therapy interventions where the patient is included in the therapy together with the relatives [79–81]., but few studies have explored the effectiveness of programs exclusively for relatives of patients with an ED diagnosis, and no studies for relatives of patients with ED-PD. Thus, this work adds knowledge about this topic and makes it possible to enhance the resources available for families of patients.

Despite the contributions of this study, we have to highlight some limitations. First, as we highlighted in a previous work [77], one of the objectives of this study is to explore the relationship between improvements in family members and improvements in patients. However, the complicated relationship between patient-parent in the ED context often makes the collaboration and assessment of patients difficult, thus affecting the results. Moreover, recruitment and program attrition problems are present, again making it difficult to reach a representative sample.

Dealing with an ED and BPD is a difficult task for relatives, who need education and skills to manage the disorder, reduce burden, and improve their quality of life [5, 6]. There is a need for studies that assess programs for these types of relatives, in order to find results that confirm their effectiveness and acceptability, as well as their clinical usefulness.

## Data Availability

It is not possible to share the data because the study is in progress. We are now at the stage of data recruitment.

## Abbreviations

ED: Eating disorders
PD: Personality disorders
FC: Family connections
FC-ED-PD: Family connections for eating disorders and personality disorders
CONSORT: Consolidated standards of reporting trials
SPIRIT: Standard protocol items: recommendations for interventional trials
TAU-O: Treatment as usual optimized
RCT: Randomized controlled trial
MANOVA: Multivariate analysis of variance
ANOVA: Analysis of variance
